# The Potential Role of Myokines/Hepatokines in the Progression of Neuronal Damage in Streptozotocin and High-Fat Diet-Induced Type 2 Diabetes Mellitus Mice

**DOI:** 10.3390/biomedicines10071521

**Published:** 2022-06-27

**Authors:** Heaji Lee, Yunsook Lim

**Affiliations:** Department of Food and Nutrition, Kyung Hee University, 26 Kyung Hee-Daero, Seoul 02447, Korea; ji3743@khu.ac.kr

**Keywords:** type 2 diabetes mellitus, sarcopenia, cognitive impairment, myokine, hepatokine, insulin resistance, energy metabolism

## Abstract

Background: Diabetes is highly prevalent, and the number of patients with diabetic sarcopenia and cognitive impairment has grown, leading to decreased quality of life. Although the exact mechanisms between sarcopenia and cognitive impairment have not been elucidated, it is speculated that muscle and liver-derived mediators might contribute to brain function. This study examined the molecular mechanisms associated with muscle-brain interaction accompanied by insulin resistance (IR) caused by aberrant energy metabolism via myokines/hepatokines in type 2 diabetes mellitus (T2DM) mice. Methods: T2DM was induced by a high-fat diet and streptozotocin injection. Behavior tests were conducted to analyze grip strength and cognitive function. Histopathological changes in skeletal muscle and brain tissue were examined by hematoxylin and eosin staining and the protein levels of biomarkers related to energy metabolism via myokines/hepatokines were measured by western blot. Results: T2DM caused peripheral and central IR. Furthermore, T2DM led to aberrant energy metabolism through the reduced fibroblast growth factor 21 dependent AMP-activated kinase (AMPK)/surtuin1/proliferator-activated receptor γ coactivator-1α pathway in T2DM. Subsequently, reduced circulating myokines/hepatokines were in accordance with their levels with hippocampal neuronal markers in T2DM mice. Accordingly, skeletal muscle (muscle strength: 2.83 ± 0.39 vs. 2.187 ± 0.51, *p* = 0.004) and brain function (PAT: 38.5 ± 57.91 vs. 11.556 ± 12.03, *p* = 0.02) impairment and morphological changes (muscle cross-sectional area: 872.43 ± 242.87 vs. 743.68 ± 169.31, *p* = 0.01; density of neurons in hippocampus: 145 ± 15.13 vs. 77 ± 35.51, *p* = 0.05; density of neurons in cortex: 138.333 ± 6.66 vs. 78 ± 17.35, *p* = 0.05) were shown in T2DM mice. In addition, the working ability demonstrated by Y-maze was positively correlated with % lean mass (*p* = 0.046, R = 0.3426). Conclusions: T2DM led to aberrant energy in skeletal muscle and brain via myokines/hepatokines. This study suggested that myokines and hepatokines might have potential roles in skeletal muscle and central metabolic functions which can mediate cognitive function in T2DM mice.

## 1. Introduction

Type 2 diabetes mellitus (T2DM) is highly prevalent and a major health problem in older adults [1]. More than 70% of adults with T2DM have difficulty performing daily physical activities, and diabetes is a strong risk factor for most senile diseases. Furthermore, T2DM is associated with an increased risk of Parkinson’s disease by 32%. In particular, emerging evidence has shown that the prevalence of sarcopenia and cognitive dysfunction is related [2,3]. Although the exact mechanisms between sarcopenia and cognitive impairment have not been elucidated, it is speculated that skeletal muscle and liver-derived mediators might contribute to brain function.

A major metabolic defect in T2DM is insulin resistance (IR) in the brain and peripheral tissue, including skeletal muscle and liver [4,5]. Impaired glucose utility causes abnormal energy homeostasis in DM patients [6]. In particular, metabolic functions in peripheral tissue, including liver, skeletal muscle, and adipose tissue interaction, are crucial for maintaining energy homeostasis [7]. Furthermore, skeletal muscle and hepatic tissue influence the release of myokines and hepatokines, respectively, which contribute to the brain function via muscle-brain/liver-brain endocrine loop [3]. 

Myokines/hepatokines are cytokines and other peptides produced and released by muscle/liver and mediate communication between other organs, including adipose tissue, liver, gut, and brain as well as within the skeletal muscle/liver itself [6]. Among them, fibroblast growth factor 21 (FGF21) acts as a myokine and hepatokine, regulating energy balance and insulin sensitivity [8]. FGF21 from the liver and skeletal muscle enters the brain tissue and activates the expression of brain-derived neurotrophic factor (BDNF) and irisin, the key modulators of neuronal neurogenesis and angiogenesis [9]. Moreover, FGF21 regulates energy metabolism by activating the AMP-activated kinase (AMPK)/surtuin 1 (SIRT1)/proliferator-activated receptor γ coactivator1α (PGC1α) pathway [10]. AMPK and SIRT1modulate cellular energy metabolism by regulating PGC1α activity and gene expression of mitochondrial enzymes [11]. PGC1α activation promotes mitochondrial replication and regulates mitochondrial biogenesis [12]. 

The enhanced AMPK/SIRT1/PGC1α pathway in skeletal muscle increases various myokines, including irisin, cathepsin-B (CTSB), and BDNF, which improve glucose homeostasis and muscle cell plasticity [11,12]. Muscle-derived CTSB, irisin, and BDNF can pass through the blood-brain barrier (BBB) in parallel with improved energy metabolism, directly enhancing the production of hippocampal irisin, BDNF, and CTSB [13,14]. Increased irisin and BDNF levels in the hippocampus are positively associated with enhanced spatial memory abilities and cognitive function in neurodegenerative disorders such as Parkinson’s and Alzheimer’s diseases [15,16,17]. Furthermore, CTSB has a neuroprotective effect by reducing amyloidogenic activity [18]. A previous study demonstrated that CTSB overexpression reduced hippocampal amyloid deposition and attenuated learning and memory loss in a mouse model of Alzheimer’s disease [19]. In this regard, multiple metabolic effects mediated by hepatic and skeletal muscle energy metabolism affect its central and peripheral metabolic functions. However, its underlying mechanism is not fully elucidated, and further studies are needed to establish the multiorgan metabolism contributing to proper physiological function in T2DM. The current study examined the molecular mechanisms of energy metabolism via myokines/hepatokines associated with neuroprotection in streptozotocin and high-fat diet-induced T2DM mice.

## 2. Materials and Methods

### 2.1. Animals and Experimental Design

**Figure 1:**
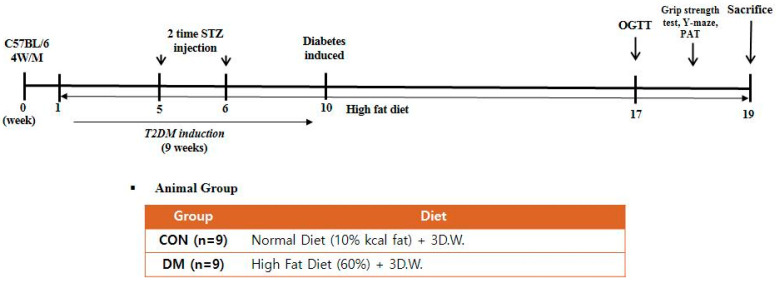
Experimental design.

Four-week-old male C57BL/6 mice (Raon Bio, Seoul, Korea) were used in the experiments. Mice were housed three per cage, as group housing promotes social exploration and provides each other social support when faced with a stressful situation. Mice were maintained under controlled temperature (22 ± 1 °C), humidity (50 ± 5%), and 12 h light and dark cycle. After acclimation for 1 week, a randomly allocated diabetic group was fed with a 60% kcal high-fat diet (D12492; Research Diets, New Brunswick, NJ, USA), whereas a non-diabetic control group (CON) was fed with a 10% kcal control diet (D12450J; matching sucrose to D12492, Research Diets, New Brunswick, NJ, USA). Food and distilled water were provided ad libitum. After four weeks, the animals were divided into two groups; CON: a normal control group and DM: a diabetes mellitus group (*n* = 9 per group). T2DM was induced by intraperitoneal (i.p.) injection of 60 mg/kg streptozotocin (Sigma-Aldrich, St. Louis, MO, USA) dissolved in citrate buffer (pH 4.5) once a week for two consecutive weeks. Non-diabetic mice were injected with only a citrate buffer. Every week after the second injection, fasting blood glucose (FBG) was measured from the mice’s tail vein using One-touch select glucometer (LifeScan, Inc., Milpitas, CA, USA). Mice with FBG > 250 mg/dL at least twice in four weeks were considered diabetic [20]. After nine weeks of diabetes induction, the CON group was fed a control diet, and the DM group was fed a high-fat diet for an additional nine weeks (Figure 1). During the experiment, body weight, food intake, and FBG levels were measured weekly. The animals were anesthetized at the end of the experiment. Blood samples were collected by heparin (Sigma Aldrich, St. Louis, MO, USA) coated syringe by cardiac puncture and were centrifuged at 845× *g* for 15 min to obtain plasma. The skeletal muscle (gastrocnemius), liver, and hippocampus tissue were frozen immediately in liquid nitrogen and stored at −80 °C until they were used for analysis. All experiments were approved by Kyung Hee University for animal welfare [KHSASP-20-060] and performed in accordance with the guidelines.

### 2.2. Muscle Function Test

A grip strength meter (Grip test package GS3 (25 N); Harvard Apparatus, Holliston, MA, USA) was used to measure forelimb grip strength at the end of the experiment. Mice were allowed to grasp the bar until they lost their grip on the metal bar. The peak pull force was recorded on a digital force transducer. The mean peak force of five trials was used for analysis.

### 2.3. Cognitive Function Test

Cognitive function tests were performed at the end of the experiment. The Y-maze apparatus consists of three dark, polyvinyl plastic arms with a 120° angle between all arms and 12 cm high walls. Mice were initially placed at the end of one arm and allowed to explore in the Y-maze freely for 8 min. The number of arm entries and the sequence of arm visits were recorded manually. The mouse consecutively entering into three different arms was defined as an actual alteration. The alternation rate (%) was calculated using the following formula: actual alternation/maximum possible alternations (total number of arm entries − 2) × 100.

A passive avoidance test (PAT) was performed in two chambers, which were square boxes of identical size (12 × 10 × 12 cm), juxtaposed as illuminated and dark. Mice were placed into the bright compartment and allowed to explore the chamber until they entered the dark compartment freely. As soon as mice crossed into the dark compartment, the door was closed, and an electrical shock (0.35 mA) was delivered for 3 s through stainless-steel rods. On the second day (after 24 h), animals were tested in the box using the same conditions without electrical shock. The time it took for the mouse to enter the dark compartment was measured, up to a maximum of 5 min.

### 2.4. Measurement of Body Composition

After the experiment, body composition was analyzed using a dual-energy X-ray absorptiometry (DXA; InAlyzer, Medikors, Seongnam, Korea). After ketamine anesthesia, each mouse was placed on the scanner bed in the prone position, with the limbs and tail stretched away from the body. Lean mass area and adipose tissue area were calculated using the manufacturer’s software [21].

### 2.5. Oral Glucose Tolerance Test (OGTT)

OGTT was performed 1 week before the end of the experiment. After overnight fasting, mice were administrated a 50% glucose solution (2 g/kg). The blood glucose level was detected at 0, 15, 30, 60, 90, and 120 min using a glucometer (OneTouch, LifeScan Inc., Malvern, PA, USA) [22]. The areas under the curve (AUC) of OGTT was calculated according to the trapezoidal rule as follows:

OGTT AUC (mg/dL·min)                     = {15 min × (BG0 min + BG15 min) × ½} + {15 min × (BG15 min + BG30 min)  × ½} + {30 min × (BG30 min + BG60 min) × ½} + {30 min × (BG60 min + BG90 min) × ½} + {30 min × (BG90 min + BG120 min) × ½}           

### 2.6. Histological Analysis

Skeletal muscle (gastrocnemius) and brain tissue were isolated and fixed in 10% buffered formaldehyde solution, dehydrated, and then embedded in paraffin wax. Tissue sections were cut into 4 µm thickness and stained with hematoxylin and eosin (H&E) by removing paraffin in xylene and rehydration in alcohol. After drying, the stained tissue sections on the glass slide were mounted with a histological mounting medium (Histomount, Atlanta, GA, USA). Tissue sections were observed using an optical microscope (Nikon ECLIPSE Ci, Nikon Instrument, Tokyo, Japan). The average cross-sectional area of skeletal muscle and the density of neurons in brain tissue were analyzed using ImageJ (National Institutes of Health, Bethesda, MD, USA) [23].

### 2.7. Hemoglobin A1c (HbA1c) % Level

HbA1c % was measured using enzyme-linked immunosorbent assay (ELISA) commercial kits (Crystal Chem, Elk Grove Village, IL, USA) according to the manufacturer’s instructions.

### 2.8. Plasma Insulin Level

Plasma insulin levels were measured using ELISA commercial kits (RayBiotech, Inc., Norcross, GA, USA) according to the manufacturer’s instructions.

### 2.9. Homeostasis Model Assessment of IR (HOMA-IR) Level

The HOMA-IR value was calculated as follows [24]:

HOMA-IR = fasting glucose (mmol/mL) × fasting plasma insulin (μU/mL)/22.5

### 2.10. Protein Extraction and Western Blot Analysis

Skeletal muscle (gastrocnemius), hippocampus, and hepatic tissue were homogenized and lysed on ice for 1 h. The lysate was centrifuged at 9000× *g* for 30 min to get cytosol extract. The pelleted nuclei remnants were resuspended in a hypertonic buffer at 9078× *g* for 20 min. The protein extract was loaded onto 5 to 15% sodium dodecyl sulfate-polyacrylamide gel electrophoresis. Proteins were transferred to a polyvinylidene fluoride membrane (Millipore, Billerica, MA, USA). The membranes were blocked with 3% bovine serum albumin for 1 h and incubated overnight at 4 °C with each primary antibody; p-Akt (Ser473), Akt, PGC1α, GLUT2, GLUT4, IRS1, FGF21, FGFR1, CTSB, BDNF, Foxo3a, Atrogin-1, Murf-1 (Santa Cruz Biotechnology, Santa Cruz, CA, USA, 1:200), PPARα, β-klotho, irisin, (Abcam, Cambridge, UK, 1:2000), LC3, p-AMPK (Thr 172), AMPK, phosphor-mammalian target of rapamycin (pmTOR), mTOR, p-IRS1 (Ser302), SIRT1, FGFR1, LC3 (Cell Signaling Technology, Danvers, MA, USA, 1:2000), proliferating cell nuclear antigen (PCNA) (Enzo Life Science, 1:1000), and α-tubulin (Sigma Aldrich, St. Louis, MO, USA). To detect primary antibodies, the relative secondary antibodies (Santa Cruz Biotechnology, CA, USA) were given to the membranes. The membranes were developed using a chemiluminescent detector (Syngene, Cambridge, MA, USA) and protein levels were quantified with GeneSnap (Syngene, Cambridge, MA, USA) [25].

### 2.11. Statistical Analysis

All data were presented as the means ± standard deviation (SD). The Shapiro–Wilk test was used to confirm the normality in statistics. The significance of differences was analyzed by the Mann-Whitney test, and *p* < 0.05 was considered statically significant. All statistical analyses were analyzed using SPSS (version 23.0 for Windows, SPSS Inc., Chicago, IL, USA). Pearson’s correlation coefficient was used to evaluate the connection between percentage of lean mass and alternation behavior on the Y-maze.

## 3. Results

### 3.1. Body Weight and Composition, Food Intake, and Glycemic Regulation in T2DM Mice

Body weight and percentage of fat of the DM group were increased compared to those of the CON group. Muscle (gastrocnemius and quadriceps) weight and % lean mass were decreased in the DM group compared to those of the CON group. The FBG level in the DM group was significantly increased compared to that of the CON group. Furthermore, plasma insulin, HbA1c %, and HOMA-IR levels in the DM group were significantly increased compared to those of the CON group (Table 1).

### 3.2. Glucose Tolerance in T2DM Mice

**Figure 2:**
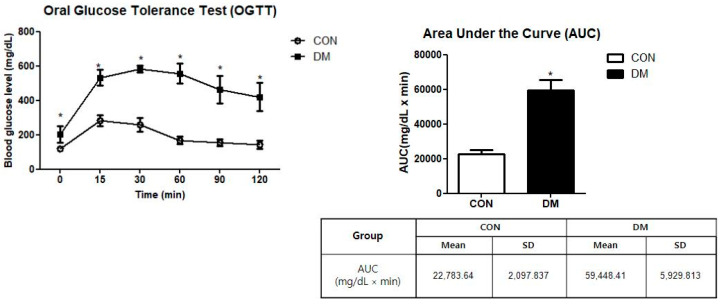
Oral glucose tolerance test in T2DM mice. All values are reported as mean and SD. The asterisk represents statistical significance of differences from * *p* < 0.05. *n* = 9 mice in each group. CON, normal control mice; DM, diabetes mellitus mice.

The DM group showed significantly higher values of FBG levels measured during OGTT than the CON group. As a result of the AUC measurement, the DM group showed significantly higher values than those of the CON group (Figure 2).

### 3.3. Skeletal Muscle Function in T2DM Mice

**Figure 3:**
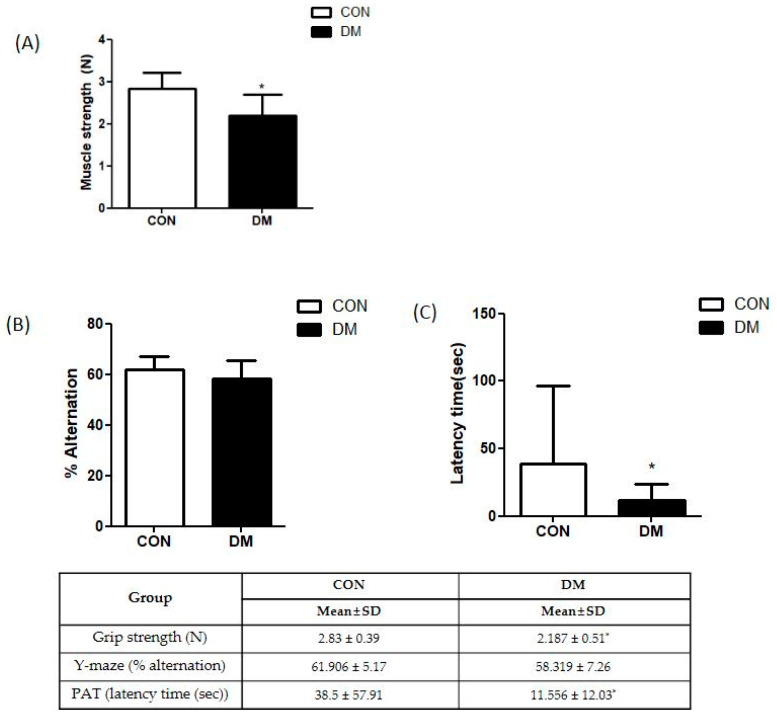
Behavior test in T2DM mice (**A**) grip strength test and (**B**) Y-maze test (**C**) passive avoidance test All values are reported as mean and SD. The asterisk represents statistical significance of differences from * *p* < 0.05. *n* = nine mice in each group. CON, normal control mice; DM, diabetes mellitus mice.

Grip strength was measured to examine skeletal muscle function. The grip strength of the DM group was significantly decreased compared to that of the CON group (Figure 3A).

### 3.4. Cognitive Function in T2DM Mice

The Y-maze and passive avoidance test (PAT) were performed to examine learning and memory abilities. There was no difference in the Y-maze test between the CON and DM groups. In PAT, latency to enter the dark compartment was significantly reduced in the DM group compared to that of the CON group after the electric shock (Figure 3B,C).

### 3.5. Correlation between % Lean Mass and Spontaneous Alternation Behavior

**Figure 4:**
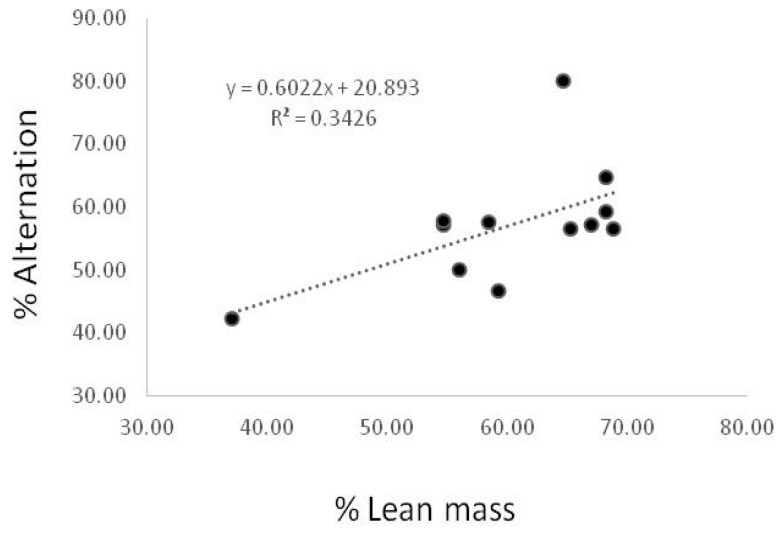
Correlation between % lean mass and spontaneous alternation behavior. Correlation between % lean and spontaneous alternation on Y maze in CON and DM group. A significant inverse relationship was observed between % lean and spontaneous alternation on Y maze in the CON and DM groups R = 0.3426, *p* < 0.05, *n* = 18).

To identify the relationship between the % lean and the spontaneous alternation behavior on Y maze, we generated a scatter plot. As shown in Figure 4, the correlation was marginally out of significance (*p* = 0.046) (Figure 4).

### 3.6. Morphological Changes of Skeletal Muscle in T2DM Mice

**Figure 5:**
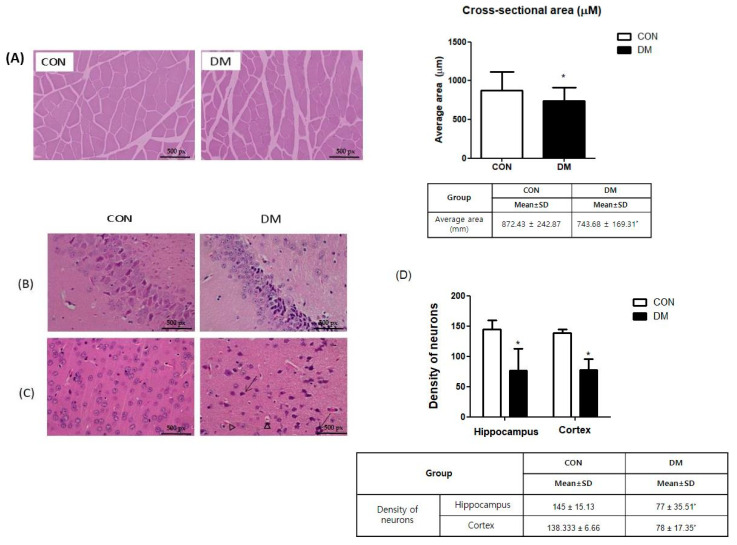
Morphological changes of (**A**) skeletal muscle and brain and (**B**) CA3, (**C**) cortex, and (**D**) density of neurons in hippocampus and cortex in T2DM mice (magnification ×200, scale bar = 500 px) Representative photos of skeletal muscle and brain section. Arrows indicate karyopyknosis of cells. Triangles indicate acidophilic necrosis. All values are reported as mean and SD. The asterisk represents statistical significance of differences from * *p* < 0.05. *n* = 9 mice in each group. CON, normal control mice; DM, diabetes mellitus mice.

In representatives of skeletal muscle tissue (gastrocnemius) stained with H&E, the average cross-sectional area of skeletal muscle in the DM group was significantly smaller than that in the CON group (Figure 5A).

### 3.7. Morphological Changes of Brain in T2DM Mice

The H&E staining demonstrated that the DM group had increased levels of nuclei pyknosis and damaged neurons compared to those of the CON group. The density of neurons in the hippocampus and cortex was decreased in the DM group compared to those of the CON group (Figure 5B–D).

### 3.8. Insulin Signaling Related Markers in T2DM Mice

**Figure 6:**
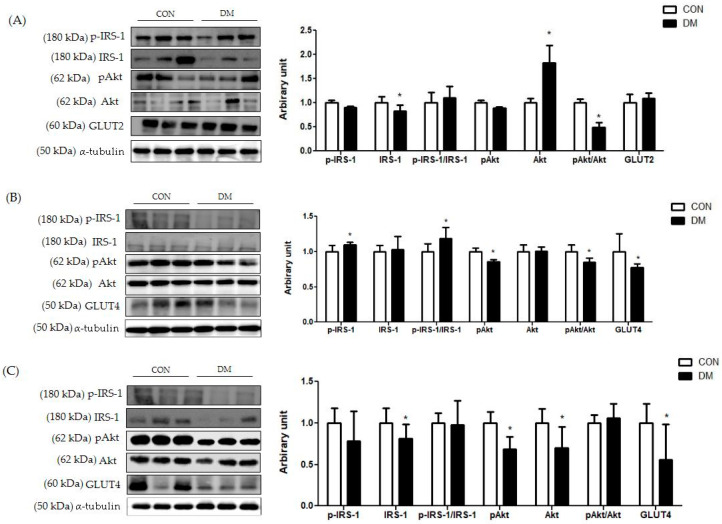
Insulin signaling in T2DM mice (**A**) liver, (**B**) skeletal muscle, and (**C**) hippocampus All values are reported as mean and SD. The asterisk represents statistical significance of differences from * *p* < 0.05. *n* = 9 mice in each group. CON, normal control mice; DM, diabetes mellitus mice.

In hepatic tissue, the protein level of *p*-IRS-1 (Ser) was not different between the groups. The protein level of IRS-1 was decreased in the DM group compared to that of the CON group. Furthermore, the ratio of pAkt/Akt was decreased in the DM group compared to that of the CON group (Figure 6A).

In skeletal muscle, the protein level of p-IRS-1 (Ser) in the DM group was increased compared to that of the CON group. The protein level of IRS-1 in the DM group was not different compared to that of the CON group. The ratio of p-IRS-1 (Ser)/IRS-1 in the DM group was increased compared to that of the CON group. Furthermore, the protein levels of pAkt and GLUT4 in the DM group were decreased compared to those of the CON group (Figure 6B).

In the hippocampus, the protein levels of IRS-1, pAkt, Akt, and GLUT4 in the DM group were decreased compared to those of the CON group (Figure 6C).

### 3.9. Energy Metabolism Related Markers with Myokines/Hepatokines and Neuronal Markers in T2DM Mice

**Figure 7:**
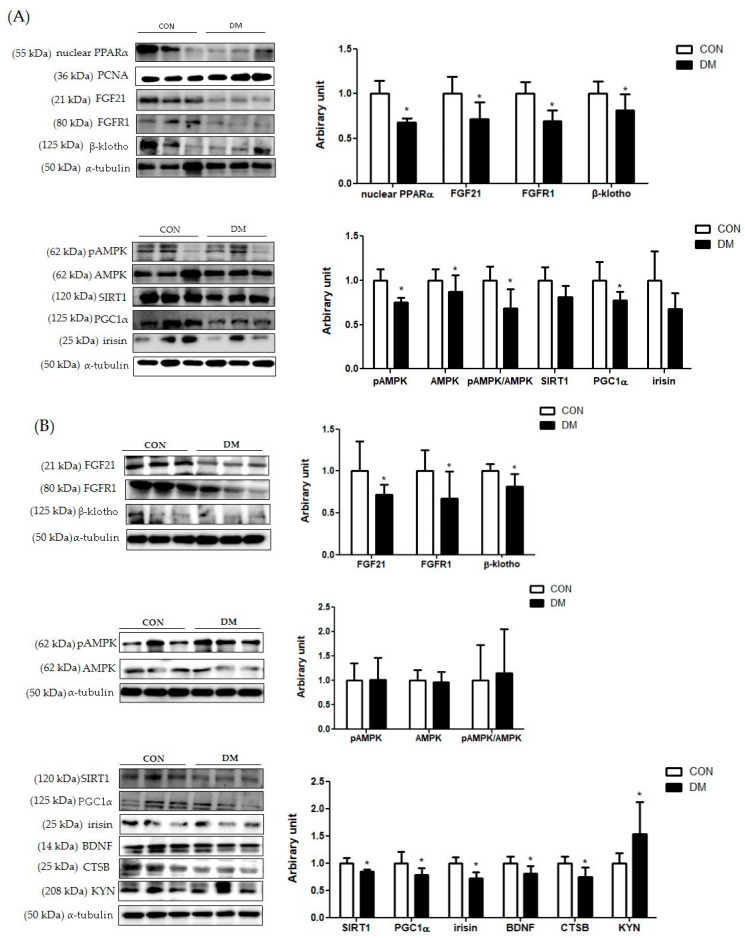

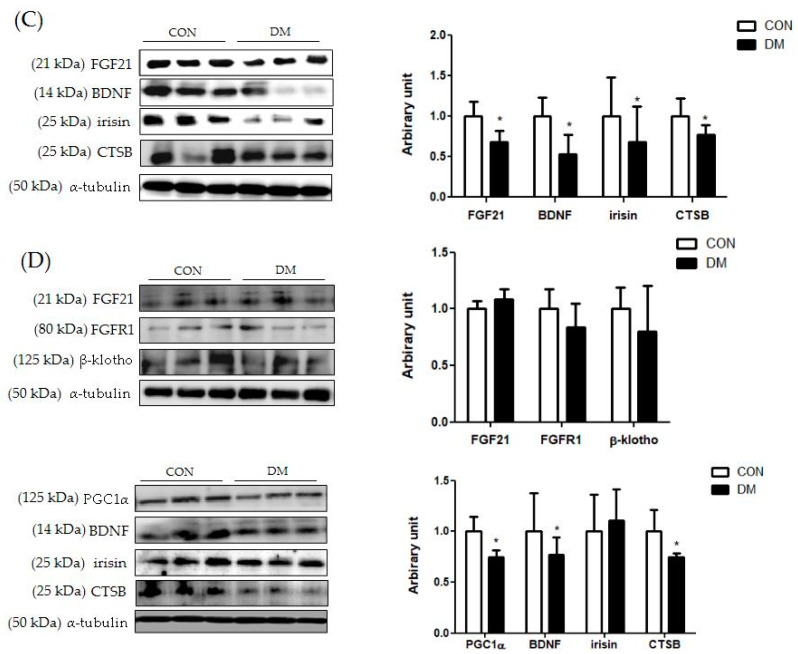
Energy metabolism related markers with myokines/hepatokines and neuronal markers in T2DM mice (**A**) liver (**B**) skeletal muscle, (**C**) plasma, and (**D**) hippocampus. All values are reported as mean and SD. The asterisk represents the statistical significance of differences from * *p* < 0.05. *n* = nine mice in each group. CON, normal control mice; DM, diabetes mellitus mice.

In hepatic tissue, the protein level of nuclear PPARα was decreased in the DM group compared to that of the CON group. Furthermore, the protein levels of FGF21, FGFR1, and β-klotho in the liver were decreased in the DM group compared to those of the CON group. The protein level of hepatic pAMPK was decreased in the DM group compared to that of the CON group. The ratio of pAMPK/AMPK in the DM group was also reduced compared to that of the DM group. The protein level of PGC1α was decreased in the DM group compared to that of the CON group. The protein levels of SIRT1 and irisin were not different between the groups (Figure 7A).

In skeletal muscle, the protein levels of FGF21, FGFR1, and β-klotho were decreased in the DM group compared to those of the CON group. The protein level of skeletal muscle AMPK was not different between the groups. The protein levels of skeletal muscle SIRT1, PGC1α, irisin, BDNF, and CTSB in the DM group were decreased compared to those of the CON group. The protein level of KYN in the DM group was increased compared to that of the CON group (Figure 7B).

In plasma, the protein levels of FGF21, BDNF irisin, and CTSB were decreased in the DM group compared to those of the CON group (Figure 7C).

In the hippocampus, the protein level of PGC1α, BDNF, and CTSB in the DM group was decreased in the T2DM mice compared to those of the CON group. The protein levels of FGF21, FGFR1, and β-klotho were not different between the groups (Figure 7D).

In summary, the protein levels of FGF21 and its receptor FGFR1 and coreceptor β-klotho in the liver and skeletal muscle were downregulated in T2DM mice. Furthermore, SIRT1/PGC1α related energy metabolism was impaired simultaneously in the liver and skeletal muscle of T2DM mice. In addition, the protein levels of myokines, including irisin, BDNF, and CTSB in skeletal muscle, were decreased in the T2DM group compared to those of the CON group. In plasma, the protein levels of FGF21, BDNF, irisin, and CTSB in the DM group were decreased compared to those of the CON group, as accordance with their levels in skeletal muscle. In the hippocampus, neuronal markers including PGC1α, BDNF, and CTSB were impaired in T2DM mice.

### 3.10. Skeletal Muscle Degradation Related Markers in T2DM Mice

**Figure 8:**
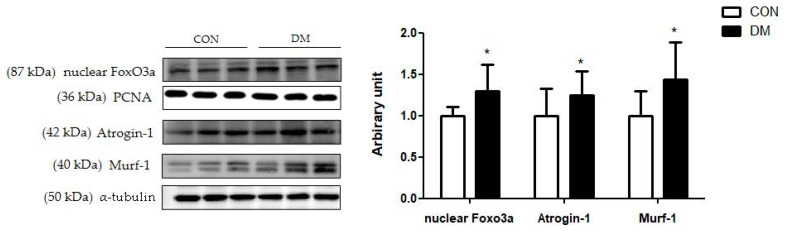
Skeletal muscle degradation related markers in T2DM mice All values are reported as mean and SD. The asterisk represents the statistical significance of differences from * *p* < 0.05. *n* = 9 mice in each group. CON, normal control mice; DM, diabetes mellitus mice.

The protein levels of skeletal muscle nuclear Foxo3a, Atrogin-1, and Mulrf-1 in the DM group were increased compared to those of the CON group (Figure 8).

### 3.11. Hippocampal Autophagy Related Markers in T2DM Mice

**Figure 9:**
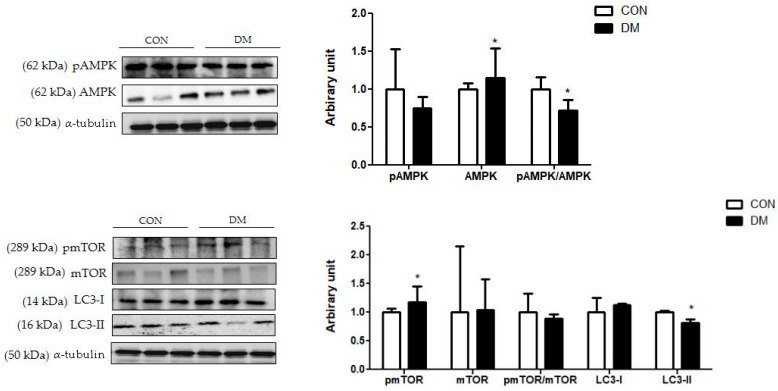
Hippocampal autophagy related markers in T2DM mice. All values are reported as mean and SD. The asterisk represents statistical significance of differences from * *p* < 0.05. *n* = nine mice in each group. CON, normal control mice; DM, diabetes mellitus mice.

The protein level of hippocampal pmTOR was increased in the DM group compared to that of the CON group. The protein level of mTOR was not different between the groups. Furthermore, the pAMPK/AMPK ratio was decreased in the DM group compared to that of the CON group. The protein level of hippocampal LC3-I was not different between the groups. The protein level of LC3-II was decreased in the DM group compared to that of the CON group (Figure 9).

## 4. Discussion

The prevalence of T2DM is increasing, and T2DM patients have multiorgan dysfunctions, which are prevalent causes of disability in the aging population [1,4,5]. Emerging evidence indicated that sarcopenia and cognitive impairment share common physiological pathways, although the molecular mechanism between them is still rudimentary. As T2DM patients are simultaneously vulnerable to complications in various tissues, it is important to understand the metabolism of multiorgans to prevent/alleviate metabolic diseases. In this study, we examined the molecular mechanisms of energy metabolism via myokines/hepatokines associated with neuroprotection in streptozotocin and high-fat diet-induced T2DM mice.

One of the risk factors for T2DM is IR accompanied by abnormal energy metabolism in the brain and peripheral tissue, including the skeletal muscle and liver [26]. Most mechanisms necessary for regulating blood glucose levels are in the peripheral tissue, but the central nervous system (CNS) also significantly influences glucose homeostasis [26]. Therefore, the regulation of energy metabolism in peripheral organs and the CNS is crucial for maintaining whole-body energy metabolism.

This study demonstrated that T2DM led to peripheral and central IR, making them dysfunctional. Impaired insulin signaling disrupted energy homeostasis [27]. In particular, aberrant energy metabolism in skeletal muscle and hepatic tissue influences the release of myokines and hepatokines, respectively, which contribute to brain function by the muscle-brain axis [3]. The “Muscle-brain axis” is introduced as myokines mediated by muscle-brain connectivity that could influence brain function. In addition, the enhanced release of endocrine factors from skeletal muscle and liver can act as a potent mediator of tissue interactions between the brain and peripheral tissue.

Among various myokines and hepatokines, FGF21 regulates a lot of pharmacological activities, such as energy metabolism, glucose and lipid metabolism, and insulin sensitivity [28]. PPARα in the liver expresses FGF21, which contributes to corticosterone release, thereby stimulating hepatic gluconeogenesis [8]. In this study, the protein levels of liver and skeletal muscle FGF21 with its coreceptor β-klotho and FGFR1 in the DM group decreased compared to those of the CON group. This means that multiple metabolic effects of the FGF21 pathway mediated by peripheral tissue were impaired in T2DM.

Furthermore, FGF21 regulates energy metabolism by activating the AMPK pathway [8]. As metabolic sensors, AMPK and SIRT1, which control the activity of the mitochondrial regulator PGC1α, are essential links in regulatory networks for metabolic homeostasis [10]. This study demonstrated that liver and skeletal muscle energy homeostasis was simultaneously impaired through the reduced FGF21-dependent energy metabolism pathway in T2DM mice. This study is significant in that it showed multiorgan aberrant energy metabolism pathways in T2DM mice. Aberrant energy metabolism with IR directly upregulates cell death pathways, including the ubiquitin proteasome system, leading to deficits in cellular homeostasis [29]. Reduced Akt activation leads to transcription factor Foxo3a, which expresses muscle-specific ubiquitin ligase causing muscle damage [30]. In this study, the protein degradation pathway in skeletal muscle was activated in the DM group, as demonstrated by increased protein levels of nuclear Foxo3a, Atrogin-1, and Murf-1 compared to those of the CON group. These results were in accordance with the morphology shown in Figure 4. T2DM decreased the average cross-sectional area of skeletal muscle, one of the pathological features of sarcopenia. In addition, skeletal muscle function was weakened, as demonstrated by the grip strength test in T2DM mice.

Damaged skeletal muscle reduces myokine production. Much evidence reported that the expression of myokines was decreased in response to energy-deficient states in skeletal muscle [31,32,33,34]. In particular, skeletal muscle-derived PGC1α, irisin, and CTSB are important for energy homeostasis not only in the peripheral but also centrally via the muscle-brain endocrine loop [13,14]. In previous studies, CTSB and irisin were released from muscle to blood during exercise, crossing the BBB and directly provoking an increase in brain PGC-1α and BDNF [35,36]. Furthermore, the application of exogenous CTSB to hippocampal progenitor cells induced BDNF transcription [12,35]. Skeletal muscle BDNF is primarily involved in autocrine and paracrine signaling to promote muscle fiber fat oxidation and potentially muscle development, and the retrograde signaling of motor neurons in the spinal cord [37]. A previous study demonstrated that BDNF levels in the brain and blood were correlated in other species, including pigs, rats, and humans [38]. In this study, the protein levels of CTSB and BDNF in skeletal muscle were decreased in the T2DM mice.

Furthermore, the current study demonstrated that T2DM simultaneously decreased the PGC1α levels in the skeletal muscle and hippocampus, which reduced the biosynthesis of the enzyme kynurenine (KYN) aminotransferase (KAT). KAT converts neurotoxic KYN into neuroprotective KYN acid, thus inhibiting toxic accumulation in the brain in T2DM mice [39]. Therefore, enhanced energy expenditure in peripheral tissue can induce hepatokines and myokines that might partially contribute to improving neurogenesis in T2DM.

In addition, this study investigated myokines and hepatokines released in circulation which can cross the BBB and have potential roles in neuronal protection. The protein levels of plasma FGF21, BDNF, irisin, and CTSB in the DM group were decreased compared to those the CON group, in accordance with their levels in skeletal muscle and hepatic tissue.

Circulating myokines/hepatokines act as powerful mediators of tissue interactions, particularly concerning the CNS. In particular, BDNF is one of the neurotrophins that protect neuronal tissue and improve CNS function [35]. Reduced hippocampal BDNF is associated with cognitive deficits in diabetic patients [40]. BDNF overexpression in the hypothalamic ventromedial nucleus increased energy expenditure [41]. Furthermore, PGC-1α can participate in energy metabolism by affecting mitochondrial dynamics, which is crucial to neuronal function. Another study demonstrated that CTSB had been considered central to antiamyloidogenic and neuroprotective activities [18]. CTSB reduced hippocampal amyloid depositions and attenuated learning and memory loss in an Alzheimer’s disease model [19]. This study demonstrated that the protein levels of CTSB and BDNF in the hippocampus of T2DM mice were impaired, which showed a similar tendency in the plasma. In addition, hippocampal FGF21 and its receptors were not different among the groups, although they were decreased in peripheral tissue. It can be suggested that brain tissue is less sensitive to energy deficiency, as it can absorb insulin better than other tissues due to the widespread expression of the insulin receptor [42].

Furthermore, autophagy is an essential cellular process in cell debris degradation to maintain cellular energy homeostasis [43]. In particular, as hippocampal neurons have poor regenerative capacity and are sensitive to various damaging substances, autophagy is particularly important and must be appropriately regulated [42]. It is widely known that AMPK stimulates autophagy by inhibiting mTOR activation, thereby increasing the LC3 expression [44]. LC3 induces the initiation of autophagosome formation. In the diabetic hippocampus, the ratio of pAMPK/AMPK was decreased and the protein level of pmTOR was increased, so that the protein level of LC3-II was decreased compared to those of the CON group. The results of this study indicated that dysregulation of autophagy impaired the balance between cell survival and death in the hippocampus of T2DM mice. In accordance with the above molecular pathways, morphological impairments were observed in the DM group. Nuclei pyknosis and damaged neurons on brain tissue were shown in the DM group. In addition, learning and memory abilities were decreased, as demonstrated by PAT in T2DM mice.

For the first time, this study demonstrated that T2DM led to abnormal energy metabolism in the liver and skeletal muscle with dysregulated hippocampal neuroprotection markers simultaneously. However, there are some limitations. In this study, the correlation between skeletal muscle and brain function could not be confirmed. Functional tests, including the hand grip strength test and PAT, were reduced, respectively, but there was no correlation between them (data not shown). Only working memory ability demonstrated by the Y-maze was positively correlated with the percentage of lean mass. It can be considered that the T2DM state in this study was not a chronic enough condition to demonstrate the functional interaction between them. In particular, brain tissue is less sensitive to peripheral IR and energy abnormality as the action of insulin is conserved in the brain [44]. Therefore, long-term studies are needed to investigate the multiorgan damage in chronic T2DM conditions. Also, although the sample size used in this study is enough to show the statistical significance, it is expected that a study with a larger sample size might provide more accurate mean values and statistical power, as individual differences can be reduced. Furthermore, the results are not enough to explain the direct effects of myokines/hepatokines on brain function. Due to the lack of effective means for crosstalk exploration, interactions between organs associated with metabolic regulation have not been studied. Although recent studies have attempted to investigate the pair of secreted molecules and their receptors to figure out specific interaction between tissues, methods to examine crosstalk networks have not been reported yet [45]. In this study, myokines/hepatokines levels in skeletal muscle and liver tissue showed a similar pattern to those of plasma and brain tissue in T2DM mice. Thus, these results demonstrated that endocrine factors derived from peripheral tissue, including skeletal muscle and liver, might have potential roles in brain function.

## 5. Conclusions

In conclusion, this study demonstrated that liver and skeletal muscle energy homeostasis was disrupted simultaneously, subsequently leading to reduced hippocampal neuroprotective markers in T2DM mice. Furthermore, this study suggested that myokines and hepatokines might have potential roles in neuroprotection in T2DM mice. Further research is needed to identify the muscle-brain interaction and clarify multiorgan targeting mechanisms as potential therapeutic targets in T2DM.

## Figures and Tables

**Table 1:** Body composition, food intake, and glycemic regulation in T2DM mice.

GROUP	CON		DM	
	Median (Min, Max)	Mean (SD)	Median (Min, Max)	Mean (SD)
Body weight (g)				
Initial body weight	26.25 (24.12, 30.09)	26.77 (1.67)	30.02 (27.25, 30.05)	30.4 (2.24) *
Final body weight	34.84 (31.7, 39.38)	35.23 (2.52)	41 (33.97, 45.414)	40.55 (3.46) *
Gain	3.26 (2.48, 3.06)	3.63 (0.96)	5.69 (1.94, 7.67)	5.32 (1.92)
% Fat	29.40 (28.05, 41.39)	31.61 (5.0)	40.34 (32.55, 59.3)	42.40 (9.07) *
% Lean	67.65 (54.67, 68.9)	65.31 (5.42)	57.24 (37.13, 65.35)	55.16 (9.56) *
Gastrocnemius weight (g/kg BW)	0.54 (0.48, 0.59)	0.54 (0.03)	0.43 (0.35, 0.48)	0.42 (0.039) *
Quadriceps weight (g/kg BW)	0.72 (0.59, 0.75)	0.71 (0.05)	0.55 (0.43, 0.61)	0.53 (0.05) *
Food Intake (g/day)	2.73 (2.32, 3.23)	2.72 (0.26)	2.53 (2.03, 3.29)	2.53 (0.34)
Fasting blood glucose level (mg/dL)	143 (119, 173)	146.36 (15.09)	263 (177,439)	291.63 (89.45) *
Plasma insulin (μU/mL)	8.66 (5.59, 18.88)	10.0 (4.07)	13.88 (7.3, 49.93)	22.49 (16.96) *
HbA1c %	4.95 (4.7, 5.4)	5.0 (0.24)	5.7 (3.2, 6.95)	5.55 (1.15) *
HOMA-IR (mmol/L × μU/mL)	2.91 (2.14, 7.5)	3.65 (1.7)	12.96 (5.06, 39.54)	16.01 (11.39) *

All values are reported as mean and SD. The asterisk represents statistical significance of differences from * *p* < 0.05. *n* = 9 mice in each group. CON, normal control mice; DM, diabetes mellitus mice.

## Data Availability

Any data generated from these studies that is not included in the present article is available upon request to corresponding author on reasonable request.
